# Guidelines for Expressing the Uncertainty of Measurement Results Containing Uncorrected Bias

**DOI:** 10.6028/jres.102.039

**Published:** 1997

**Authors:** Steven D. Phillips, Keith R. Eberhardt, Brian Parry

**Affiliations:** National Institute of Standards and Technology, Gaithersburg, MD 20899-0001; Boeing Corporation, Seattle, WA 98124-2207

**Keywords:** bias, error, expanded uncertainty, measurement, uncertainty

## Abstract

This paper proposes a method to extend the current ISO *Guide to the Expression of Uncertainty in Measurement* to include the case of known, but uncorrected, measurement bias. It is strongly recommended that measurement results be corrected for bias, however in some situations this may not be practical, hence an extension of the *Guide* is proposed to address this special situation. The method keeps with the spirit of the *Guide* in maintaining the link between uncertainty and statistical confidence. Similarly, the method maintains the transferability of one uncertainty statement to be included as a component in another uncertainty analysis. The procedure involves modifying the calculation of the expanded uncertainty, allowing it to become asymmetric about the measurement value. The method is compared to other alternative procedures, and an illustration of how it affects tolerance zones is presented.

## 1. Introduction

Recently, the ISO *Guide to the Expression of Uncertainty in Measurement* (the *Guide*) [1], and the associated NIST adaptation [2], have described a unified convention for expressing measurement uncertainty. Application of the *Guide* has extended beyond calibration and research laboratories and into the industrial domain of manufactured products. At the factory floor level the recommended (and strongly preferred) practice of correcting for all known bias effects may not be economically possible due to such factors as limited instrumentation, operator training, and the large throughput of measurements. Since the *Guide* does not deal directly with the situation where a known measurement bias is present but is uncorrected, we propose a simple convention to extend the *Guide’s* procedures to address this special case. Uncorrected measurement bias may arise in situations where applying a correction for a known measurement bias would be costly, but increasing the measurement uncertainty to allow for the uncorrected bias would still result in an acceptable uncertainty statement. Initially, it might seem paradoxical to be aware of a measurement bias but fail to correct for it; however, such situations are rather common. For example, the user of an automated instrument may know a bias occurs under certain measurement situations, and be unable to modify the behavior of the instrument. Since “paper and pencil” corrections to each measurement value can be time consuming and error prone, particularly under high measurement throughput situations, it may be more economically reasonable to simply account for this bias by enlarging the uncertainty value that is attached to every measurement result.

**Fig. 1:**
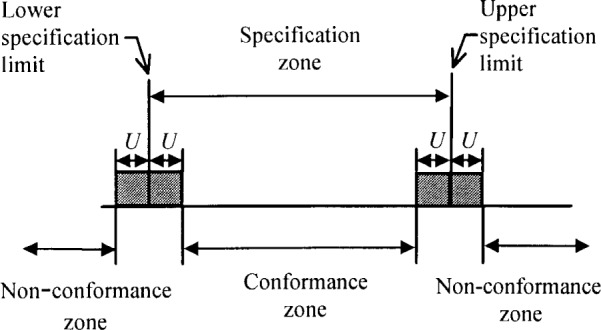
The functional specification of a part and the corresponding inspection zones; *U* is the expanded uncertainty of the measurement. Parts are accepted if the measurement result is within the conformance zone.

A few examples of measurements that include uncorrected bias are now presented to illustrate the situation. A manufacturer of a precision positional indicator may know that all the indicators produced read approximately 0.5 % too high, with only a small variation of 0.1 % (standard deviation) between indicators. This is within the required 1 % relative uncertainty specification for the indicators and satisfies the customer’s needs. It may be expensive and difficult to adjust the manufacturing process to reduce the 0.5 % bias, or even to apply a 0.5 % correction to each unit; consequently, it is easier to subsume this bias into the uncertainty statement. Another example may be an automated instrument that is sensitive to some slowly varying parameter such as temperature, atmospheric pressure, humidity, etc. The instrument may lack a sensor to input this parameter and the user may be “locked out” of the software which records the measurement results; hence an automated correction cannot be performed to account for this systematic bias. However, the user may be able to specify the upper and lower acceptance limits, i.e., the conformance zone, for the measurement; see Fig. 1. If this bias is sufficiently small, it may be economically sensible to subsume it into the uncertainty statement. Doing so will alter the expanded uncertainty, and hence modify the conformance zone.

The *Guide* primarily addresses the situation in which all known biases have been corrected, which is the recommended practice. However, Appendix F of the *Guide* does briefly discuss the situation of uncorrected measurement bias. It is our intention to extend this procedure and to provide examples of its implementation. Our motivation for this effort includes the observation that some industrial practitioners, in an effort to be consistent with the *Guide*, have included the bias as an ordinary uncertainty source which is added in the usual root-sum-of-squares (RSS) manner. This has the undesirable effect of incorrectly stating the expanded uncertainty, because the bias is added in an RSS manner and is multiplied by the coverage factor.^1^ Hence, the relationship between measurement confidence and the expanded uncertainty is broken, as illustrated in detail later. Since many parts are accepted or rejected on the basis that the measurement results demonstrate conformance with the part specification [3] (see Fig. 1), it is important not to misstate the uncertainty (or confidence) associated with the measurement.

This document describes a convention to account explicitly for uncorrected measurement bias in an uncertainty statement. We believe any method to include measurement bias in the uncertainty statement should have the following desirable properties.

1. The final quoted uncertainty must be greater than or equal to the uncertainty that would be quoted if the bias was corrected. Underestimating the uncertainty indicates an invalid uncertainty statement. Similarly, excessively overestimating the uncertainty indicates a poorly constructed uncertainty statement.
2. The method must reduce to the method given by the *Guide* when the bias correction is applied.
3. For any coverage factor and any magnitude of bias, the level of confidence for the expanded uncertainty should be at least the level obtained for the case of corrected bias, e.g., if the distribution of the values that could reasonably be assigned to the measurand is Gaussian, then *k* = 2 should imply at least 95 % confidence.
4. The method should be transferable so that both the uncertainty and the bias from one result can be used as components in another uncertainty statement.
5. The method should be simple and inexpensive to implement.

## 2. Recommendations for Measurements Involving Uncorrected Bias

As described in the *Guide*, measurement results should be corrected for bias, and the uncertainty in the bias correction should be included as a contribution to the combined standard uncertainty. However, when correcting for the measurement bias is not practical, it still should be accounted for explicitly in the uncertainty statement. In our proposed approach, a complete uncertainty statement includes the combined standard uncertainty (computed as if the measurement result was to be corrected for the bias), an explicit statement of the (signed) bias value, and an expanded uncertainty which includes the effect of the bias term.

The usual method of using the expanded uncertainty *U*, for a measurement result *y* which has an unknown (“true”) value of the measurand *Y*, is to produce an uncertainty interval (with the level of confidence determined by the coverage factor) given by: *y* – *U* ≤ *Y* ≤ *y* + *U.* Consequently, the measurement result is often stated as: *y* ± *U*.

In the case where the result is corrected for a bias *δ*, a similar uncertainty interval can be constructed for the corrected measurement result *y*_cor_ = (*y* − *δ*) given by: *y*_cor_ − *U* ≤ *Y* ≤ *y*_cor_ + *U*. This is equivalent to the uncertainty interval of: *y* – (*U* + *δ*) ≤ *Y* ≤ *y* + (*U* – *δ*). Consequently, the measurement result could be stated as: $y\left\{ \begin{array}{l} +(U-\delta )\\ -(U+\delta ) \end{array}\right.$.

This can lead to the unfortunate possibility that one of the uncertainty limits may become negative, e.g., if the bias is positive and *δ* > *U* then the upper uncertainty limit will be negative. This may confuse practitioners, particularly when constructing diagrams such as Fig. 1; consequently, we propose the additional requirement that the uncertainty limits be greater than or equal to zero for all values of *δ*, which always maintains non-negative uncertainty limits at a cost of a somewhat wider uncertainty interval.

**Fig. 2:**
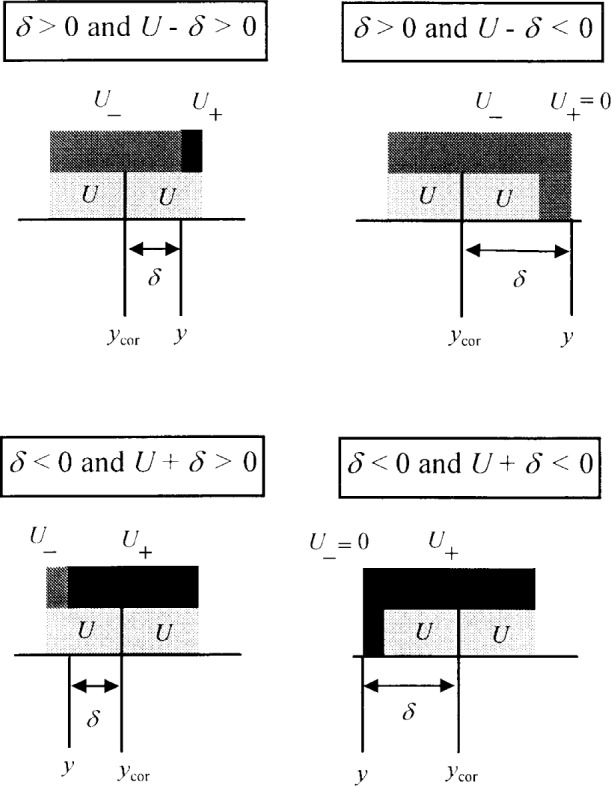
Uncorrected measurement result *y*, having bias *δ* (*δ* > 0), shown with expanded uncertainties *U_+_* (black) and *U*_−_ (dark gray) (top). The corresponding corrected measurement result *y*_cor_, is also shown together with the usual expanded uncertainty *U* (light gray) (bottom). The same situation with *δ* < 0.

Hence, for a measurement result *y* which includes an uncorrected bias *δ*, the value of the measurand *Y* is estimated by the following uncertainty interval where *U* is the usual expanded uncertainty that would be calculated if the measurement had been corrected for bias; see Fig. 2. An uncertainty interval in the presence of uncorrected bias is given by:

$$\begin{array}{l} y-U_{-}\leq Y\leq y+U_{+}\\ \text{or equivalently}\;Y=y\left\{ \begin{array}{l} +U_{+}\\ -U_{-} \end{array}\right. \end{array}$$

$$\begin{array}{l} \text{where}\;U_{+}=\{\begin{array}{ll} U-\delta & \text{if}\;U-\delta >0\\ 0 & \text{if}\;U-\delta \leq 0 \end{array}\\ \text{and}\;U_{-}=\{\begin{array}{ll} U+\delta & \text{if}\;U+\delta >0\\ 0 & \text{if}\;U+\delta \leq 0 \end{array}. \end{array}$$

Note that a large bias may result in a one sided uncertainty interval, e.g., if *δ* > *U* then *U*_−_ = *U* + *δ* and *U*_+_ = 0. (One could propose a symmetric uncertainty interval, with the expanded uncertainty given by the larger of *U*_+_ or *U*_−_, but this further reduces the conformance zone with no additional economic benefit.)

When computing an uncertainty statement for cases where there are several sources of uncorrected bias, biases are algebraically added together (explicitly accounting for the sign of the bias). The resulting net bias is stated together with the combined standard uncertainty. Occasionally, the case may arise where multiple sources of uncertainty have bias and these biases are not independent. To avoid “double counting” the bias sources, the degree of overlap of the biases is estimated and this amount is subtracted from the bias summation. The uncertainty in the overlap correction is added in a RSS manner to the combined standard uncertainty. Finally, we point out that the expanded uncertainty must be re-computed if the coverage factor is changed, and in particular, that *U*_±_ (*k* = 2) ≠ 2 × *U*_−_ (*k* = 1).

**Fig. 3:**
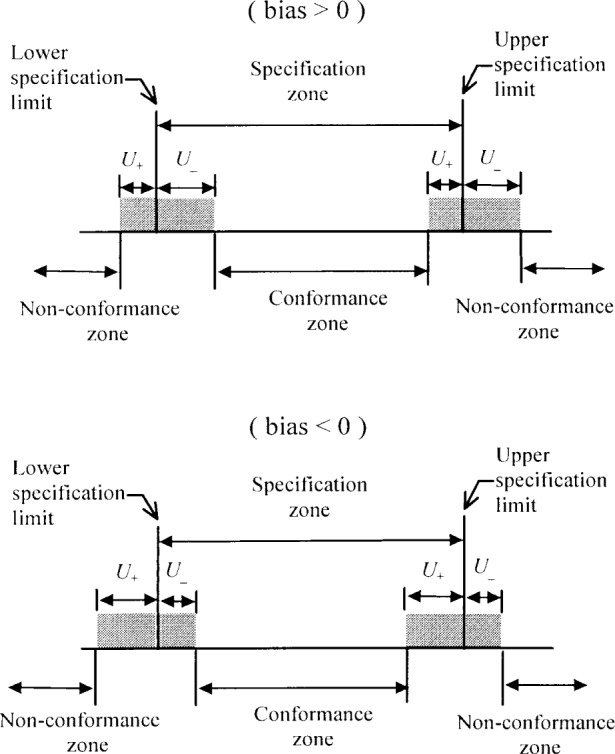
An example of the functional specification of a part and the corresponding inspection zones for a measurement result with uncorrected bias; *U*_+_ and *U*_−_ are the expanded uncertainties which include the effects of measurement bias (top: bias > 0, bottom: bias < 0). Parts are accepted if the measurement result is within the conformance zone.

The proposed approach is recommended for its simplicity and utilitarian value (see Fig. 3), even though it can somewhat overestimate the uncertainty. However, for a given coverage factor the corresponding level of confidence will be at least as high as would be the case if the bias had been corrected.

## 3. Comparison With Other Methods of Combining Uncorrected Bias

We compare our proposed method of treating uncorrected bias with two other procedures which have been proposed to address this problem. The first procedure treats the uncorrected bias as another uncertainty source and simply sums it in an RSS manner with the usual combined standard uncertainty; we denote this method as RSS*u*_c_. The second method sums the bias in a RSS manner with the usual expanded uncertainty; we denote this as RSS*U*. In contrast our proposed method algebraically sums the signed bias with the expanded uncertainty (unless the bias is large), so we denote our method as SUM*U*. The three methods are shown below.

RSS*u*_c_ Method: $Y=y\pm U_{RSSu_{c}}$
where $U_{RSSuc}=k\sqrt{u_{c}^{2}+\delta^{2}}$
RSS*U* Method: $Y=y\pm U_{RSSU}$
where $U_{RSSU}=\sqrt{k^{2}u_{c}^{2}+\delta^{2}}$
SUM*U* Method: $Y=y\left\{ \begin{array}{l} +U_{+}\\ -U_{-} \end{array}\right.$
where $U_{+}=\{\begin{array}{ll} ku_{c}-\delta & \text{if}\;ku_{c}-\delta >0\\ 0 & \text{if}\;ku_{c}-\delta \leq 0 \end{array}$
and $U_{-}=\{\begin{array}{ll} ku_{c}+\delta & \text{if}\;ku_{c}+\delta >0\\ 0 & \text{if}\;ku_{c}+\delta \leq 0 \end{array}$.

**Fig. 4:**
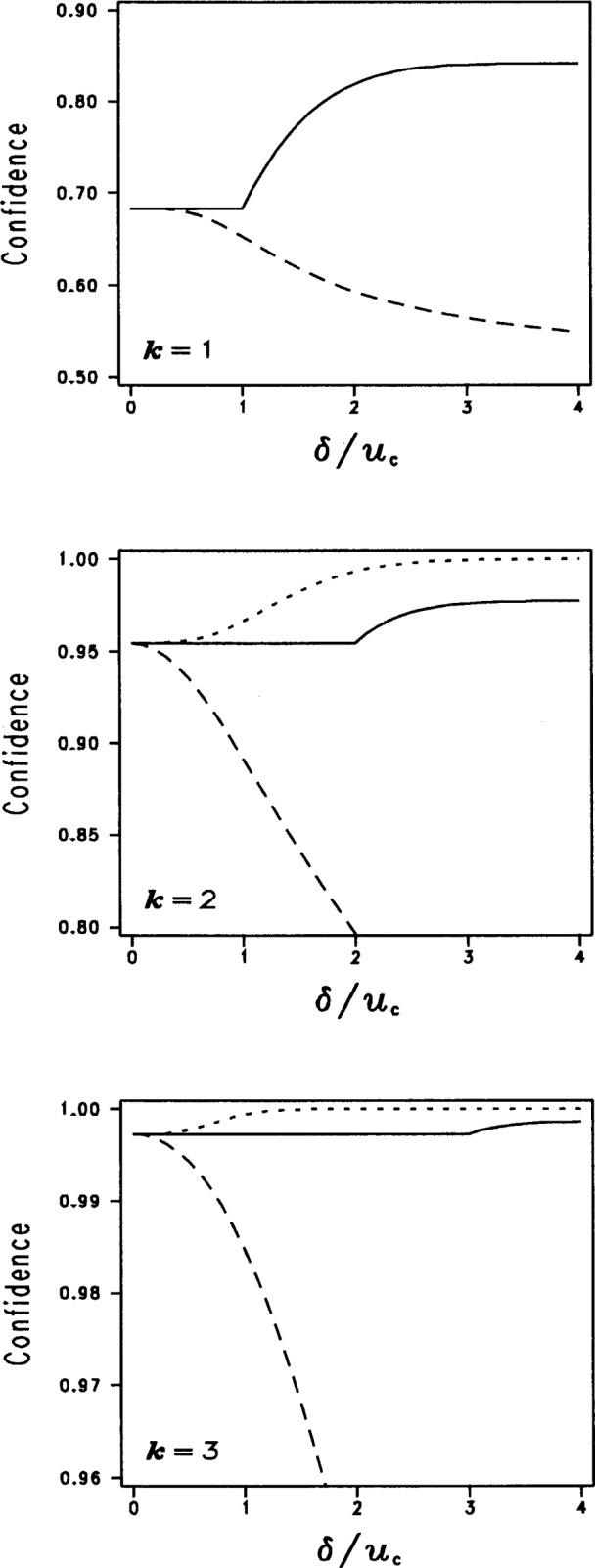
Comparison of the actual achieved confidence levels resulting from three methods of incorporating uncorrected bias in uncertainty intervals. The methods are the proposed SUM*U* method (solid line), the RSS*u*_c_ method (dotted line) and the RSS*U* method (dashed line). (In the case *k* = 1, RSS*u*_c_ = RSS*U*.)

Figure 4 illustrates some important differences between the three methods. The three plots display the actual statistical confidence of the three methods versus the magnitude of the uncorrected bias for coverage factors of *k* = 1, 2, and 3. Gaussian (normal) distributions are assumed in all cases. For example, in the *k* = 2 case, ideally we would like to maintain a 95 % (strictly speaking, 95.44 %) confidence for all values of the uncorrected bias. Our proposed method (SUM*U*) maintains this confidence until the ratio of the bias to the combined standard uncertainty becomes larger than the coverage factor. For such large bias values, the SUM*U* method produces uncertainty intervals that are slightly conservative (larger than necessary to produce valid 95 % confidence levels.) The RSS*u*_c_ method, on the other hand, can produce uncertainties that are considerably larger than necessary. For example, with *k* = 2 and a bias twice as large as the combined standard uncertainty (*δ*/*u*_c_ = 2), the actual achieved confidence level of the interval is nearly 100 %, rather than the nominal 95 % (see Fig. 4.) Although this overstatement of the uncertainty is not necessarily disastrous, it can come at the significant cost of consuming most of the part tolerance zone, i.e., specification zone, as we will soon describe. In contrast, the RSS*U* method seriously understates the true uncertainty. For example, with a coverage factor of *k* = 2 and an uncorrected bias twice as large as the combined standard uncertainty (*δ*/*u*_c_ = 2), the uncertainty interval is under-sized to the extent that the actual achieved confidence is less than 80 %, which falls significantly short of the nominal 95 % confidence level.

**Fig. 5:**
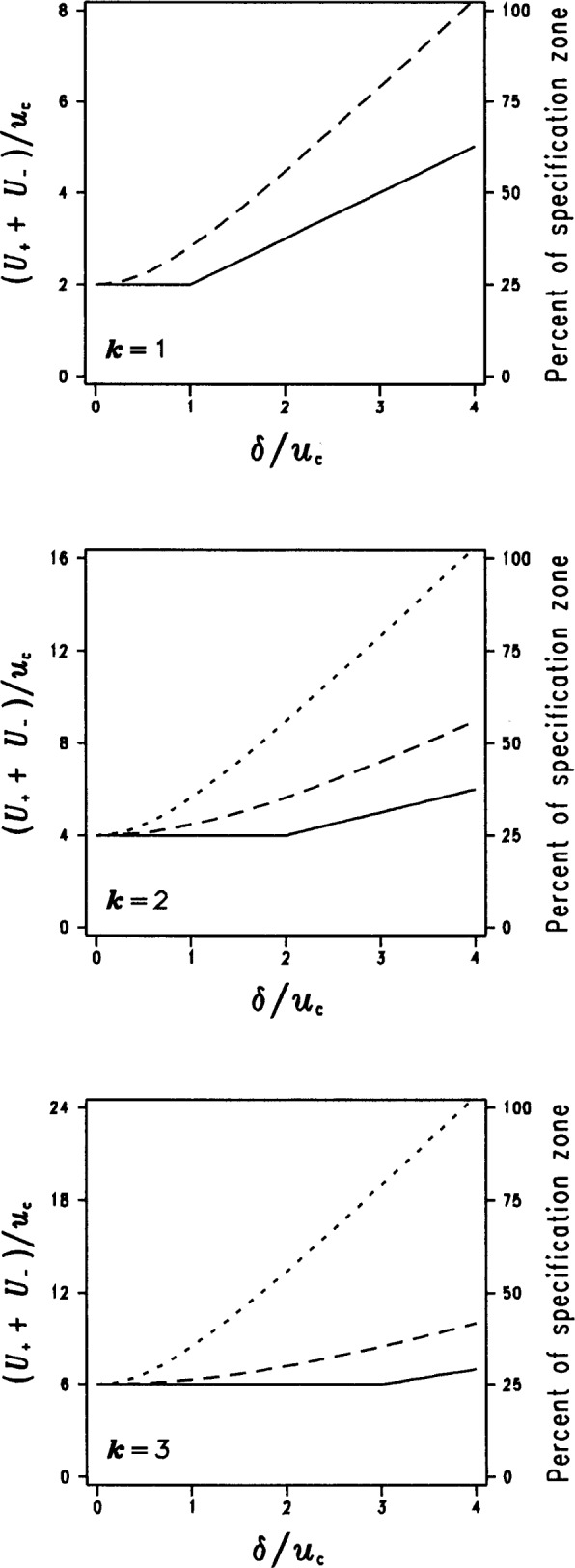
Comparison of uncertainty interval lengths for three methods of incorporating uncorrected bias in uncertainty intervals. The methods are the proposed SUM*U* method (solid line), the RSS*u*_c_ method (dotted line) and the RSS*U* method (dashed line). The scale on the right-hand axis of the plots assumes that the value of the expanded uncertainty represents a gauging ratio of 4:1 in the case of zero bias (*δ* = 0). (In the case *k* = 1, RSS*u*_c_ = RSS*U*.)

The three plots in Fig. 5 display the relative sizes of the expanded uncertainty interval for each of the three methods as a function of the magnitude of the uncorrected bias, for the coverage factors *k* = 1, 2, and 3. The scale of the ordinate on the left hand side of the plots is defined as the full width of the uncertainty interval divided by the combined standard uncertainty, and hence the ratio would be equal to 2 *k* (where *k* is the coverage factor) if the bias had been corrected. As seen in the figure, the SUM*U* method consistently produces the smallest expanded uncertainty interval of any of the methods for all values of bias and coverage factors.

An example of how the size of the expanded uncertainty interval might impact the user is shown on the right hand ordinate. This axis describes the percentage of the specification zone that is consumed by the expanded uncertainty interval for the somewhat typical case where the ratio of the specification zone to expanded uncertainty interval zone (if the bias had been corrected) is 4:1. (Obviously, the right hand ordinate axis is numerically correct only for the particular example of a gauging ratio of 4:1; other ratios, while having qualitatively similar behavior, would have different percentages of the specification zone consumed by the expanded uncertainty interval.) The plot illustrates that the SUM*U* method consumes the smallest percentage of the specification zone compared to the other two methods. For example, in the *k* = 2 case, and for an uncorrected bias equal to four times the combined standard uncertainty (*δ*/*u*_c_ = 4), the SUM*U* method would consume 37.5 % of the specification zone (compared to 25 % if the bias had been corrected), while the RSS*U* and RSS*u*_c_ methods consume 56 % and over 100 %, respectively.

Figures 4 and 5 illustrate that, of the three methods described, our proposed method (SUM*U*) offers a significant advantage in reducing the impact on the user of the uncorrected bias when subsumed into the uncertainty statement. This method always maintains an actual level of confidence equal to or greater than the nominal confidence corresponding to the coverage factor in use. While our examples are based on the Gaussian distribution, the SUM*U* method retains this relationship (between *k* and the confidence level) for any distribution shape because the resulting interval always contains at least the same interval as would be covered by the corrected result. Furthermore, of the three methods discussed, the SUM*U* method minimizes the percentage of the specification zone consumed by the expanded uncertainty interval. In addition, the method avoids negative expanded uncertainties, which could be confusing to the user when determining the conformance zone (as shown in Fig. 3).

## 4. Examples of Uncertainty Statements Containing Uncorrected Bias

The following examples should clarify the procedure for expressing measurement uncertainty in the presence of uncorrected bias. We use the same terminology as the *Guide* when referring to the evaluation of the bias, i.e., Type A corresponds to any valid statistical method for treating the data and Type B corresponds to a bias evaluated by other means. For each example we give a worked numerical case to illustrate the procedure; in these examples all expanded uncertainties are evaluated with a coverage factor of *k* = 2. These examples are contrived to illustrate the procedure of accounting for uncorrected bias and are not designed to describe the subtleties of creating an uncertainty statement; consequently many uncertainty sources may have been omitted or simplified.

### 4.1 Example 1: One Type A bias

Consider a measurement result *y*, having a constant bias of estimated magnitude *δ*_1_. Assume that *δ*_1_ is assessed directly by repeated measurements of a reference standard having a combined standard uncertainty of *u*_ref_. Specifically, suppose *δ*_1_ is evaluated as the average deviation from the reference standard’s calibrated value found from *N*_1_ measurements. Let the experimental standard deviation of the *N*_1_ measurements be *s*; the standard uncertainty of the estimated bias is then $s/N_{1}^{1/2}$. The combined standard uncertainty of the measurement result is given below, where *u*_1_ accounts for the combination of all other uncertainty sources not directly associated with the bias. Note that *u*_1_ already includes the repeatability of the measurement, i.e., the standard deviation *s*, since this source of uncertainty is always present and is unaffected by the fixed bias. The combined standard uncertainty is the same quantity that would be determined if the measurement had been corrected for the bias. Note that the expanded uncertainty is treated asymmetrically and the results depend on the sign of the bias. In this example *δ*_1_ < 0 and *ku*_c_ + *δ*_1_ > 0.

$$\begin{array}{l} u_{c}={\left(u_{1}^{2}+\frac{s^{2}}{N_{1}}+u_{\text{ref}}^{2}\right)}^{\frac{1}{2}}\\ \text{Bias}=\delta_{1}\\ U_{+}=ku_{c}-\delta_{1}\\ U_{-}=ku_{c}+\delta_{1} \end{array}$$

The uncertainty interval is given by *y* –*U*_−_≤*Y*≤*y*+*U*_+_. Equivalently the result can be stated with the expanded uncertainty as $Y\left\{ \begin{array}{l} +U_{+}\\ -U_{-} \end{array}\right.$.

Numerical case: Suppose that a measuring machine designed to inspect parts of length 100 mm is checked with a reference standard having a combined standard uncertainty of *u*_ref_ = 1.5 μm. A total of 15 measurements are recorded having an experimental standard deviation of 3.0 μm with the mean result 4.0 μm smaller than the calibrated value of the reference standard, i.e., the bias is negative. From previous work it is known that all other uncertainty sources combined yield a standard uncertainty of 5.0 μm, i.e., *u*_1_ = 5.0 μm. Therefore:

$$\begin{array}{l} u_{c}={\left(5.0^{2}+\frac{3.0^{2}}{15}+1.5^{2}\right)}^{\frac{1}{2}}\text{μm}=5.3\;\text{μm,}\\ \text{Bias}=-4.0\;\text{μm}\\ U_{+}=2u_{c}-(-4.0)\;\text{μm}=14.6\;\text{μm}\\ U_{-}=2u_{c}+(-4.0)\;\text{μm}=6.6\;\text{μm,} \end{array}$$

and the measurement result *y* can be stated with the expanded uncertainty as: $y\left\{ \begin{array}{l} +14.6\;\text{μm}\\ -\;6.6\;\text{μm} \end{array}\right.$.

### 4.2 Example 2: One Type B bias

For some measurements, the bias might be estimated rather than directly measured. For example, length measurements made on the factory floor often are not corrected back to the standard temperature of 20 °C. Hence, the uncorrected thermal expansion represents a measurement bias. Suppose the factory floor temperature varies between 20 °C and 30 °C, about an estimated mean of 25 °C. The estimated magnitude of the bias is given by *δ*_2_ (*δ*_2_ > 0) which accounts for the length deviation due to the 5 °C mean uncorrected thermal expansion. The variability of the temperature can be described by a uniform distribution of full width 10 °C, i.e., by a standard uncertainty of 2.9 °C which, when multiplied by the appropriate coefficient of thermal expansion, gives rise to the corresponding standard uncertainty *u*_temp_. The combined standard uncertainty and expanded uncertainty are given below, where *u*_2_ would be the combined standard uncertainty for the measurement if the measurement had been corrected back to 20 °C. (The value of *u*_2_ includes the uncertainties in the temperature measurements, the uncertainties in the thermal expansion coefficient, and other effects.) In this example *δ*_2_ > 0 and *ku*_c_ – *δ*_2_ > 0.

$$\begin{array}{l} u_{c}={\left(u_{2}^{2}+u_{\text{temp}}^{2}\right)}^{\frac{1}{2}}\\ \text{Bias}=\delta_{2}\\ U_{+}=ku_{c}-\delta_{2}\\ U_{-}=ku_{c}+\delta_{2} \end{array}$$

The uncertainty interval is given by *y* –*U*_−_≤*Y*≤*y*+*U*_+_. Equivalently the result can be stated with the expanded uncertainty as $y\left\{ \begin{array}{l} +U_{+}\\ -U_{-} \end{array}\right.$.

Numerical case: Suppose a measuring machine that inspects parts of length 100 mm has *u*_2_ = 7.0 μm, and the machine’s scale has a thermal expansion coefficient of 9 (μm/m)/°C, and the part under inspection has a thermal expansion coefficient of 22 (μm/m)/°C. Then the differential thermal expansion is 22 (μm/m)/°C – 9 (μm/m)/°C = 13 (μm/m)/°C, corresponding to an average bias of 13(μm/m)/°C×5 °C×0.100 m=6.5μm. The standard uncertainty associated with the variation in the temperature (modeled as a uniform distribution) is given by *u*_temp_ = 13 (μm/m)/°C × 0.100 m × 2.9 °C= 3.8 μm. Therefore:

$$\begin{array}{l} u_{c}={\left(7.0^{2}+3.8^{2}\right)}^{\frac{1}{2}}\text{μm}=8.0\;\text{μm,}\\ \text{Bias}=+6.5\;\text{μm}\\ U_{+}=2u_{c}-6.5\;\text{μm}=9.4\;\text{μm}\\ U_{-}=2u_{c}+6.5\;\text{μm}=22.4\;\text{μm,} \end{array}$$

and the measurement result *y* can be stated with the expanded uncertainty as: $y\left\{ \begin{array}{l} +\;9.4\;\text{μm}\\ -22.4\;\text{μm} \end{array}\right.$.

### 4.3 Example 3: Combination of independent biases

An uncertainty statement consists of two uncertainty sources given by those of examples 1 and 2, which are assumed to be independent. The resulting uncertainty statement is given below. Note that *δ*_3_ is the sum of the two biases, and that we assume *δ*_3_ > 0 and *ku*_c_–*δ*_3_ > 0; *u*_c1_ and *u*_c2_ are the combined standard uncertainties from examples 1 and 2, respectively.

$$\begin{array}{l} u_{c}={\left({u_{c}}_{1}^{2}+{u_{c}}_{2}^{2}\right)}^{\frac{1}{2}}\\ \text{Bias}=\delta_{3}=\delta_{1}+\delta_{2}\\ U_{+}=ku_{c}-\delta_{3}\\ U_{-}=ku_{c}+\delta_{3}. \end{array}$$

The uncertainty interval is given by *y* –*U*_−_≤*Y*≤*y*+*U*_+_. Equivalently the result can be stated with the expanded uncertainty as $y\left\{ \begin{array}{l} +U_{+}\\ -U_{-} \end{array}\right.$.

Numerical case: Using the values given in examples 1 and 2 we find

$$\begin{array}{l} u_{c}={\left(5.3^{2}+8.0^{2}\right)}^{\frac{1}{2}}\text{μm}=9.6\;\text{μm,}\\ \text{Bias}=+2.5\;\text{μm}\\ U_{+}=2u_{c}-2.5\;\text{μm}=16.7\;\text{μm}\\ U_{-}=2u_{c}+2.5\;\text{μm}=21.7\;\text{μm,} \end{array}$$

and the measurement result *y* can be stated with the expanded uncertainty as: $y\left\{ \begin{array}{l} +16.7\;\text{μm}\\ -21.7\;\text{μm} \end{array}\right.$.

### 4.4 Example 4: Combination of independent and dependent biases

The measuring instrument described by the uncertainty statement of Example 3 is modified by an accessory that does not add variability but produces an additional bias *δ*. This bias is assessed by repeated measurements, i.e., found from *N*_4_ measurements of a second (independent) reference standard (having a combined standard uncertainty of *u*_ref2_). The measurements collectively have an experimental standard deviation *s* (this is the same standard deviation found in example 1), and a mean value differing from the calibrated value by *δ*, with *δ* < 0 and *δ* < *δ*_1_ < 0. It is estimated that between 30 % to 50 % of the bias estimated by *δ* is already accounted for in *δ*_1_. To avoid double counting, 0.4 *δ* (which is the best estimate of the overlap, i.e., the average of 30 % and 50 % = 40 % = 0.4) is subtracted from the bias summation. A standard uncertainty of $0.1\delta /\sqrt{3}$, corresponding to a uniform distribution (from 0.3 *δ* to 0.5 *δ* with half width 0.1 *δ*), accounting for the uncertainty of the bias overlap is added in an RSS manner to the other standard uncertainties. We assume the total net bias *δ*_4_ > 0 and *ku*_c_ – *δ*_4_ > 0, as shown below.

$$\begin{array}{l} u_{c}={\left({u_{c}}_{1}^{2}+{u_{c}}_{2}^{2}+u_{\text{ref}2}^{2}+\frac{s^{2}}{N_{4}}+\frac{{\left(0.1\delta \right)}^{2}}{3}\right)}^{\frac{1}{2}}\\ \text{Bias}=\delta_{4}=\delta_{1}+\delta_{2}+\delta -0.4\delta =\delta_{1}+\delta_{2}+0.6\delta \\ U_{+}=ku_{c}-\delta_{4}\\ U_{-}=ku_{c}+\delta_{4}. \end{array}$$

The uncertainty interval is given by *y* –*U*_−_≤*Y*≤*y*+*U*_+_. Equivalently the result can be stated with the expanded uncertainty as $y\left\{ \begin{array}{l} +U_{+}\\ -U_{-} \end{array}\right.$.

Numerical case: The additional bias *δ* = –2.0 μm is evaluated as the mean of 10 measurements using a reference standard with combined standard uncertainty *u*_ref_*_2_* = 1.0 μm. Using the values given in the previous examples, we find

$$\begin{array}{l} u_{c}={\left(5.3^{2}+8.0^{2}+1.0^{2}+\frac{3.0^{2}}{10}+\frac{0.2^{2}}{3}\right)}^{\frac{1}{2}}\text{μm}=9.7\text{μm,}\\ \text{Bias}=+1.3\;\text{μm}\\ U_{+}=2u_{c}-1.3\;\text{μm}=18.1\;\text{μm}\\ U_{-}=2u_{c}+1.3\;\text{μm}=20.7\;\text{μm,} \end{array}$$

and the measurement result *y* can be stated with the expanded uncertainty as: $y\left\{ \begin{array}{l} +18.1\;\text{μm}\\ -20.7\;\text{μm} \end{array}\right.$.

## Footnotes

1 An inaccurate method for calculating an expanded uncertainty can lead to the uncertainty being either overstated or understated, depending on the values of the bias, combined standard uncertainty, the coverage factor, and the shape of the distribution. When the uncertainty is overstated (i.e., is too large), the nominal confidence level claimed will be smaller than it should be to properly describe the given uncertainty. Conversely, if the uncertainty is understated (i.e., is too small), the nominal confidence level claimed will be larger than it should be.
